# Feeding ecology of elasmobranch fishes in coastal waters of the Colombian Eastern Tropical Pacific

**DOI:** 10.1186/1472-6785-7-8

**Published:** 2007-09-18

**Authors:** Andrés F Navia, Paola A Mejía-Falla, Alan Giraldo

**Affiliations:** 1 Fundación Colombiana para la Investigación y Conservación de los Tiburones y Rayas, SQUALUS. Carrera 64 A No 11A-53, Cali, Colombia, USA; 2 Grupo de Investigación en Ecología Animal, Sección de Zoología, Departamento de Biología, Universidad del Valle. A.A. 25360, Cali, Colombia, USA

## Abstract

**Background:**

Stomach contents of 131 specimens of five elasmobranch species (*Mustelus lunulatus*, *Dasyatis longa*, *Rhinobatos leucorhynchus*, *Raja velezi *and *Zapteryx xyster*) caught in the central fishing zone in the Pacific Ocean of Colombia were counted and weighed to describe feeding habits and dietary overlaps.

**Results:**

Twenty-one prey items belonging to four major groups (stomatopods, decapods, mollusks and fish) were identified. Decapod crustaceans were the most abundant prey found in stomachs. The mantis shrimp *Squilla panamensis *was the main prey item in the diet of *M. lunulatus*; tiger shrimp *Trachypenaeus *sp. was the main prey item in the diet of *Rhinobatos leucorhynchus *and *Raja velezi*, and Penaeidae shrimp were the main prey items in the diet of *Z. xyster*. Furthermore, fish were important in the diet of *Raja velezi, Z. xyster *and *D. longa*. The greatest diet breadth corresponded to *Z. xyster *whereas *M. lunulatus *was the most specialized predator. Finally, four significant diet overlaps between the five species were found, attributable mainly to Squillidae, Penaeidae and Fish.

**Conclusion:**

Shrimps (Penaeidae and stomatopods) and benthic fishes were the most important food types in the diet of the elasmobranch species studied. Diet breadth and overlap were relatively low. Determination of food resource partitioning among the batoid species studied was not possible. However, we identified partitions in other niche axes (time of feeding activity and habitat utilization). It is possible to assume that diffuse competition could be exceeding the biunivocal competition among the studied species. Therefore, this assemblage would have a strong tendency to trophic guild formation.

## Background

Elasmobranch fishes are among the top predators in the marine environment and thus play an important role in marine ecosystems, potentially regulating, through predation, the size and dynamics of their prey populations [1]. An understanding of competitive and predatory processes is thus necessary to gain insight into the role of predators in influencing niche, community and food web structure, and ultimately ecosystem dynamics [2]. In this sense, the theory about resource partitioning is frequently attributed to competitive or cooperative interactions. This approximation predicts that spatial or temporal partitioning may increase the tolerance of niche overlap, and may reduce the pressure of competition among coexisting species [3,4].

Several studies have evaluated the influence of resource partitioning in teleost fishes to explain reduction in competition potential [5-7]. These authors reported a positive relationship between habitat partitioning and the magnitude of diet overlap or competitive interactions, when the interacting species had the same diet preferences. Furthermore, Ross [8] found that the first resource that is fractioned is food, followed by habitat. Despite the ecological importance of elasmobranch fishes for the marine ecosystem, resource partitioning and competitive exclusions in this taxonomic group are poorly understood.

A substantial amount of data on the diet of different elasmobranch species has been reported to date. Elasmobranch fishes are often typified as opportunistic predators, with a wide trophic spectrum that includes plankton to marine mammals. In general, oceanic elasmobranch species feed on squid and big fishes [9,10], whereas the coastal and benthic species feed on crustaceans, mollusks and small or juvenile fishes [11-13]. A few species feed on other elasmobranchs, birds, reptiles or marine mammals [14,15]. Ontogenetic variation in diet is well known [16,17], with a strong tendency to ingest larger and more mobile animals with increasing size.

Research on trophic relationships among sympatric species of elasmobranchs is scarce and results vary among studies [18-20]. Varying overlap values between coexisting species, successive sizes of a same species, or even between sexes have been reported. Moreover, the effect of habitat and feeding time on the diet of sympatric species is very poorly known [21-23]. In the Eastern Pacific Ocean of Colombia (EPOC) the study of elasmobranch diet is in its infancy [24-26]. The existing studies have only considered three of 87 elasmobranch species reported for the area [27]. Furthermore, no research has addressed trophic interactions among sympatric fish species in coastal or oceanic environments.

Therefore, the object of this study was to quantify and compare the diet and trophic interactions of coastal elasmobranch species from the Eastern Tropical Pacific of Colombia, and to suggest possible mechanisms for their coexistence.

## Results

### Feeding activity

**Figure 1:**
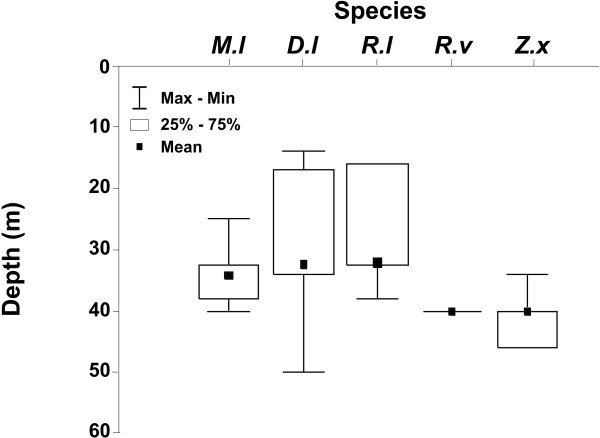
Bathymetric distribution of the elasmobranch species studied. (M.l) is *Mustelus lunulatus*, (D.l) is *Dasyatis longa*, (R.l) is *Rhynobatos leucorhynchus*, (R.v) is *Raja velezi *and (Z.x) is *Zapteryx xyster*.

A total of 131 specimens of five species (*Mustelus lunulatus*, *Dasyatis longa, Rhinobatos leucorhynchus*, *Raja velezi *and *Zapteryx xyster*) were analyzed. All specimens were captured in shallow waters (between 15 and 60 m depth). Significant differences in the bathymetric distribution of the species were found (KW-H (4,162) = 105.16, p << 0.0001), with *Zapteryx xyster *and *Raja velezi *found in narrower depth ranges and under 35 and 40 m, respectively. *Dasyatis longa *and *Rhinobatos leucorhynchus *were associated with the shallowest depths (Figure 1). *Mustelus lunulatus*, *D. longa *and *Rhinobatos leucorhynchus *were captured mainly during diurnal bottom trawling, while *Z. xyster *were captured mainly during nocturnal bottom trawling. *Raja velezi *was captured only at night. Only *R. leucorhynchus *and *Z. xyster *showed significant differences in hourly feeding activity (Table 1). We did not carry out analyses of ontogenetic shifts in diet because we did not capture individuals from all sizes intervals.

**Table 1:** Sampling data of elasmobranchs caugth

		Capture (n)			Size (cm)	
Species	n	Diurnal	Nocturnal	p	Min	Max
*Mustelus lunulatus*	42	15	16	0.8202	55	125
*Dasyatis longa*	21	19	5	0.8906	106	210
*Rhinobatos leucorhynchus*	24	10	18	**0.0017**	37	67
*Raja velezi*	13	0	15	-	48	80
*Zapteryx xyster*	31	3	51	**0.0353**	27	66

Sampling data of elasmobranchs caugth in the Eastern Tropical Pacific Ocean of Colombia and range size of these species. *n*: number of individuals captured. p: p-value of K-W test.

### Diet composition

The proportion of empty stomachs varied widely among species, with a minimum vacuity index of 9.5% in *M. lunulatus *and a maximum vacuity index of 47.6% in *D. longa *(Table 2). The main prey item of *Mustelus lunulatus *were stomatopods from the family Squillidae (%N = 73.2%, %W = 36.1%, and occurrence 78.6%) (Table 3), with *Squilla panamesis *being the main prey item (92.8 %IRI). An additional twelve prey categories were identified in the stomachs of *M. lunulatus*, which feed opportunistically on shrimps, crabs and gasteropods in low percentages.

**Table 2:** Feeding activity of captured elasmobranchs

Specie	n	Es	VI	χ_n_	CI_χn_	χ_w_	CI_χw_
*Mustelus lunulatus*	42	3	9.5	2.5	1.9 – 3.1	8.9	7.8 – 11
*Dasyatis longa*	21	10	47.6	0.9	0.3 – 1.2	1.1	0.3 – 1.9
*Rhinobatos leucorhynchus*	24	5	20.8	1.8	1.1 – 2.5	1.3	0.6 – 2.0
*Raja velezi*	13	4	38.8	1.0	0.3 – 1.7	6.3	0.2 – 12.4
*Zapteryx xyster*	31	11	35.5	0.9	0.5 – 1.3	1.1	0.6 – 1.6

Feeding activity of elasmobranch captured in the Eastern Tropical Pacific Ocean of Colombia. Number of stomachs analyzed (*n*), Empty stomachs (*Es*), Vacuity Index (*VI*), mean number of prey per stomach (*x*_*n*_), 95% Confidence Interval for χ_*n *_(*CIxn*), mean weight of prey per stomach (*x*_*w*_), 95% Confidence Interval for χ_*w *_(*CIxw*).

**Table 3:** Diet composition of captured elasmobranchs

	***M. lunulatus***				***D. longa***				***R. leucorhynchus***				***R. velezi***				***Z. xyster***			
	% O	% N	%W	% IRI	% O	% N	%W	% IRI	% O	% N	%W	% IRI	% O	% N	%W	% IRI	% O	% N	%W	% IRI
CRUSTACEA																				
STOMATOPODA																				
Squillidae					14.28	58.30	4.48	54.87	4.16	2.85	0.31	0.31								
*Squilla panamensis*	61.90	62.90	28.10	92.77													12.90	18.20	26.00	20.83
*Squilla parva*	14.28	9.27	5.95	4.01																
*Pseudosquilla similis*	2.38	1.03	2.07	0.12																
**Total Stomatopoda**	**78.56**	**73.20**	**36.10**	**96.90**	**14.28**	**58.30**	**4.48**	**54.87**	**4.16**	**2.85**	**0.31**	**0.31**					**12.90**	**18.20**	**26.00**	**20.83**
DECAPODA																				
Portunidae									8.33	8.57	0.47	1.79								
*Euphylax *sp.	2.38	1.03	0.40	0.05																
*Portunus asper*	4.76	3.09	7.32	0.78																
*Callinectes toxotes*	2.38	1.03	0.40	0.05																
*Hepatus *sp.	4.76	2.06	0.75	0.21																
Brachiura																	3.20	4.54	0.59	0.56
Penaeidae									25.00	31.30	10.30	24.82					19.40	27.3	31.4	41.89
*Pennaeus occidentales*	7.14	2.06	0.75	0.23																
*Trachypenaeus *sp.	4.76	2.06	0.40	0.49					33.30	45.70	30.30	60.44	15.38	46.20	45.70	29.57				
Palaemonidae	2.38	9.27	4.11	0.49																
Hipiddae	7.14	4.12	2.20	0.71	4.76	8.33	15.70	6.98									3.20	4.54	2.09	0.74
**Total Decapoda**	**35.70**	**24.70**	**16.50**	**3.01**	**4.76**	**8.33**	**15.70**	**6.98**	**66.63**	**85.60**	**41.70**	**87.05**	**15.38**	**46.20**	**45.70**	**29.57**	**25.80**	**36.40**	**34.10**	**43.19**
MOLLUSCA																				
GASTEROPODA																				
Buccinidae	2.38	1.03	0.03	0.04																
*Agaronia testacea*					4.76	8.33	7.62	4.59												
*Distorsio decussata*					4.76	8.33	7.62	2.71												
BIVALVIA																				
Terebridae	2.38	1.03	0.16	0.04																
**Total Mollusca**	**4.76**	**2.06**	**0.19**	**0.08**	**9.52**	**16.70**	**7.92**	**7.30**												
FISHES																				
Fish					9.50	16.70	36.80	31.05	12.50	11.40	30.90	12.64	38.46	38.50	37.40	61.17	16.10	27.30	14.60	24.81
Batrachoididae													15.38	15.40	13.40	9.26				
Cynoglossidae																	9.70	18.20	13.70	11.16
**Total Fishes**					**9.50**	**16.70**	**36.80**	**31.05**	**12.50**	**11.40**	**30.90**	**12.64**	**53.84**	**53.90**	**50.80**	**70.43**	**25.80**	**45.50**	**28.30**	**35.97**
Digested material			47.20				35.10				27.10				3.52				11.40	

Prey items observed in stomachs of five species of elasmobranchs captured in the Eastern Tropical Pacific Ocean of Colombia. %O is frequency of occurrence, %N is percentage by number, %W is percentage by weight

In *D. longa *stomatopods were the most frequent prey item (14.3%) and had the highest percent by number (58.3%), whereas fishes had the highest percent by weight (36.8%) (Table 3). Digested material was 35.1% and vacuity index was 47.6%. The main prey items were Squillidae (54.9% %IRI) and fishes (31.1%IRI). The diet of this species also included decapods and gastropods.

In *Rhinobatos leucorhynchus *the most frequent (66.6%), abundant (85.6%) and with highest percent by weight (41.7%) prey item was decapods, following by fishes (12.5, 11.4 and 30.9% respectively) (Table 3). The main prey item was *Trachypenaeus *sp. (60.4%IRI). However, stomatopods and crabs (Portunidae) were also found in the diet of this species. The vacuity index was low (20. 8%).

Only two prey items were identified in *R. velezi*, fishes and decapods. Fishes were the most frequent item (53.8%), and also the most abundant by number (53.9%) and weight (50.8%), and collectively (70.4% IRI). Among the fishes, only Batrachoididae could be identified (Table 3).

Six prey categories were identified in stomach contents of *Z. xyster *(Table 3). Fishes and penaid shrimp occurred most frequently (> 16%) and were the most abundant by number (27.3% each), however shrimp were the most abundant by weight (31.4%). For this species decapods were the most important prey item with 43.2 %IRI.

### Diet breadth and overlap

**Figure 2:**
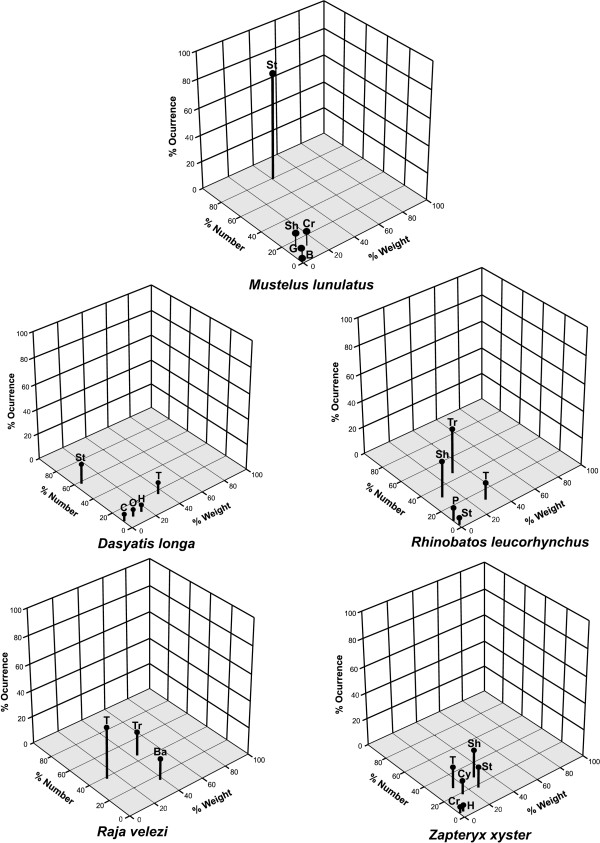
Three dimensional graphical representation of the relative importance of prey in the diet of five elasmobranch species: percentage by number **(%N)**, percentage by weight **(%W) **and frequency of ocurrence **(%O)**. Prey items are: St) Stomatopods, Cr) Crabs, Sh) Shrimps, B) Bivalvia, G) Gasteropoda, T) Teleost, O) Olividae, H) Hippidae, C) Cymatidae, Tr) *Trachypenaeus*, P) Portunidae, Ba) Batrachoididae, Cy) Cynoglossidae.

Use of a three-dimensional graphical representation of the diet [28] facilitated the description of feeding styles of the five species studied (Figure 2). The diet of *M. lunulatus *was specialized, with most individuals consuming *Squilla panamensis*, although this species also feed on twelve other prey items. In contrast, the diet of *Z. xyster *was more heterogeneous and generalized because fishes, decapods and stomatopods were consumed by the majority of individuals. These results were confirmed by Levin's breadth index, which yielded the greatest diet breadth for *Z. xyster*, while *M. lunulatus *was the most specialized predator (Table 4).

**Table 4:** Diet breadth of elasmobranchs caugth

		Percentage by number (*%N*)	
Species	N	*B*_*i*_	*B*_*A*_

*Mustelus lunulatus*	13	2.40	0.12
*Dasyatis longa*	5	2.57	0.39
*Rhinobatos leucorhynchus*	5	3.05	0.51
*Raja velezi*	3	2.60	0.53
*Zapteryx xyster*	6	4.57	0.71

Levins' measure of niche breadth (*B*_*i*_) and standarized *B*_*i *_(*B*_*A*_) calculated with percentage by number (*%N*) data. N: total number of prey categories used.

Following Zares & Rand's [29] criteria, four biologically significant diet overlaps were established among the studied species (Table 5). A significantly high overlap was found between *M. lunulatus *and *D. longa*, which shared shrimps of the family Squillidae. Significant overlaps were also found between *Rhinobatos leucorhynchus *and *Raja velezi*, and *R. leucorhynchus *and *Z. xyster*, which shared Penaeidae, and between *Raja velezi *and *Z. xyster *which shared fish and Penaeidae. The random overlap hypothesis was rejected (p = 0.010), suggesting low overlap and low partitioning of resources [30].

**Table 5:** Niche overlap of elasmobranchs caugth

**Species A**	**Species B**				
	
	*M. lunulatus*	*D. longa*	*R. leucorhynchus*	*R. velezi*	*Z. xyster*
*M. lunulatus*	1.000	-	-	-	-
*D. longa*	**0.930**	1.000	-	-	-
*R. leucorhynchus*	0.097	0.072	1.000	-	-
*R. velezi*	0.035	0.208	**0.729**	1.000	-
*Z. xyster*	0.343	0.515	**0.603**	**0.935**	1.000

Niche overlap between five species of elasmobranch caugth in the Eastern Tropical Pacific Ocean of Colombia. Values were calculated using the Pianka measure, indicating the extent to which the diet of Species A overlaps with the diet of Species B. Significant diet overlap (>0.6).

## Discussion

The composition of diets suggests that all elasmobranchs studied are species that feed on epibenthic invertebrates (mainly stomatopods and decapods) and fish. This result is highly associated with the source of the samples analyzed, i.e., elasmobranchs captured in bottom trawl fishery. Predators found during this activity, feed mainly on benthic infauna (prey species living in the sediment), epibenthic fauna (prey species living on the surface of the sediment), benthic prey (prey species living on the bottom) or demersal prey (prey species living near the bottom but not linked to it). Therefore, the results of this research are restricted to elasmobranchs that feed on prey species living on the sediment surface or inside the sediment. However, our results contribute significantly to increase the knowledge on the feeding ecology of the other 138 species of fishes coexisting in the same habitat [31], with similar food and space requirements.

*Mustelus *species have been reported to feed mainly on crustaceans and fish [18,25,26,32], although cephalopods would also be important in their diets [33]. Despite the large number of taxa found in the stomach contents of *M. lunulatus*, one species of stomatopod accounted for most of the prey consumed (stomatopoda = %IRI = 96.9). Therefore, *M. lunuatus *in coastal waters of the Colombian Eastern Tropical Pacific have a specific dietary preference. Besides Ellis et al. [18], this is the only other report of feeding specialization in *Mustelus*, a genus that has been classified as opportunistic polyphagous [34,35].

The diet of *Raja velezi *in the neritic zone of the Eastern Pacific Ocean of Colombia showed a low number of prey items in compared with other studies [20,36,37] that found polychaetes, copepods, amphipods, Myscidacea, stomatopods, cephalopods and bivalves. However, the preference of *R. velezi *to feed on decapods and fishes detected in this study was similar to that reported for *R. naevus *[18], *R. clavata *[37], *R. radiata *[38], and *R. brachyura*, *R. montagui *and *R. eglanteria *[39], which also feed upon decapods or fish. Although *R. velezi *is a deep-water ray with a widespread distribution, our results are the first description of the diet of this species in the Tropical Pacific Ocean. *Rhinobatos leucorhynchus *has been identified as a crustacean predator, with preference for penaeid shrimp [22,34,40,41], although portunid crabs have also been reported as alternative prey [42].

Very little is known of the diet of *Dasyatis longa *and *Zapteryx xyster *and this study is the first record on the diet of these species. Similar to other benthic species *Dasyatis longa *feed on crustacean decapods (stomatopods) and fishes. The preferences for these dietary items have also been reported for *Dasyatis americana *in the Caribbean Sea [43]. *Zapteryx xyster *showed the widest feeding spectrum of all elasmobranch studied. This species included stomatopods, decapods and fishes in its diet and, was the most generalist species. Bornatowski et al. [44] reported similar results for *Z. brevirostris *from South Brazil.

Macpherson [45] and Cortés [46] suggested that the relatively big body size of elasmobranch makes it easy for them to expand their feeding spectrum, preying simultaneously on the pelagic and benthic communities. Our study shows that diet breadth was reduced, and prey item distribution by number and weight were similar. Moreover, all studied elasmobranch fed on three main items: Squillidae, Penaeidae and fish. This can be explained by the benthic habits and similarity of sizes and dental structure between *M. lunulatus *and *D. longa*, as well as between *Rhinobatos leucorhynchus, Raja velezi and Z. xyster*.

The complete dietary overlap detected between *M. lunulatus *and *D. longa*, their similar geographical distribution in the study area, and their diurnal activity, suggest that these species could show a competitive exclusion trend for food resources [47].

However, the dietary overlap would be reduced when the deep distribution is considered because *M. lunulatus *occupies deep waters and *D. longa *shallow waters. In contrast the dietary overlap detected between *Rhinobatos leucorhynchus*, *Raja velezi *and *Z. xyster *would be significant, since these species have morphological (size and position mouth) and behavioral similarities.

The dietary overlap between *Rhinobatos leucorhynchus *– *Raja velezi *and *R. leucorhynchus *– *Z. xyster*, based on the presence of *Trachypenaeus *shrimp and fishes, could be compensated by differential diel feeding activity. *Rhinobatos leucorhynchus *feed mainly during the day whereas *R. velezi *and *Z xyster *feed manly at night. Furthermore, 89% of *Rhinobatos *whose stomachs were analyzed were caught at a deep of 15–30 m, whereas all *Raja *and *Zapteryx *specimens were caugth at a depth of 40 m. In contrast, the dietary overlap between *R. velezi *and *Z. xyster*, species with similar feeding activity and bathymetric distribution, suggests that they share only a fraction of their feeding niche, protecting a portion as an uncontested space refuge [47]. In this sense, mantis shrimp would be the feeding refuge of *Z. xyster*. Dietary overlap between *Z. xyster *and *R. velezi *was fish unidentified and decapods; however this food category was so indeterminate that the evidence of effective dietary overlap is weak. Furthermore, this wide category of prey item overestimate dietary overlap and underestimate niche breadth.

Values of overlap based on simulations suggest that the species studied showed low dietary overlap (mean = 0.44). In this study only five species of elasmobranchs were examined. In the study area at least another 12 species of elasmobranchs [31,48] and six species of fishes [31] that share morphological and ecological characteristics with the studied species have been reported. However, all these species and the elasmobranchs that we studied would conform apackaging throughout a feeding axis, and the temporal dynamics of this packaging could be determined by: (i) variety and productivity of available resources, (ii) short-term and long-term environmental variability, and (iii) niche overlap [49,50]. Therefore, if the packaging is modulated by resource availability [50] then the shrimp trawl fishery in the neritic zone of Eastern Pacific of Colombia has severely affected the natural availability of prey for elasmobranch communities [51,52], affecting their structure.

## Conclusion

Shrimps (penaeid and stomatopods) and benthic fishes were the main component of the diet of the elasmobranchs studied. Diet breadth and overlap were relatively low. Determination of food resource partitioning among the batoid species studied was not possible. However, we identified partitions in other niche axes (time of feeding activity and habitat used). It is possible to assume that diffuse competition could be exceeding the biunivocal competition among the studied species. Therefore, this assemblage would have a strong tendency to trophic guild formation.

## Methods

### Study area and sampling

**Figure 3:**
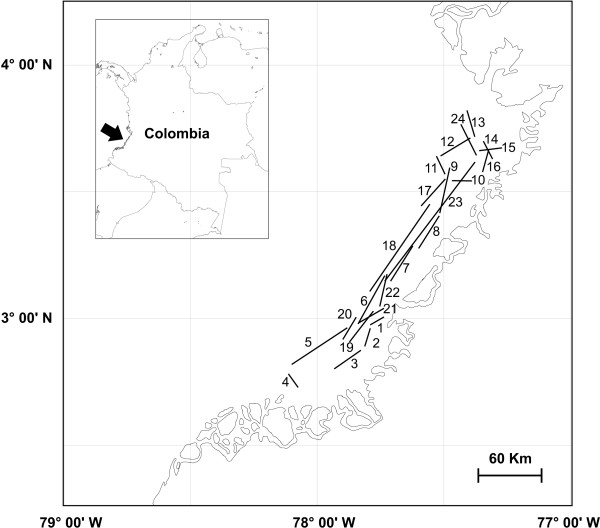
Eastern Pacific Ocean of Colombia (EPOC) showing the central fishing area. Each number represents one trawling episode.

Stomachs were collected from elasmobranchs caught by bottom trawling with commercial shrimp nets (2 m mouth, 50 mm mesh size), during three cruises in the central fishing area of Eastern Pacific of Colombia (Fig. 3). Twenty four trawls were made over the continental shelf (5–60 m depth) from the bottom prawn trawler vessel *Arraijan *from June to November 2001.

The total length of captured elasmobranch was recorded and the stomachs of all specimens were extracted on board, fixed in 10% formalin, labeled, bagged and transported to the laboratory. Once in the laboratory an incision was made along the longitudinal axis and the stomach contents were emptied into a 1000 ml plastic bucket for rinsing and sorting. Food items were identified to the lowest taxonomic level possible. Numbers and weights (to the nearest 0.1 g) of food items were recorded after the items were dried with blotting paper to remove surface moisture.

### Numerical analysis

The quantitative importance of each prey group in the diet of elasmobranch species was estimated by using the frecuency of occurrence (%O), percentage by number (%N) and percentage by weight (%W) of prey items in stomachs [28] as:

%O=nN×100%N=NnNp×100%W=PpPt×100
 MathType@MTEF@5@5@+=feaafiart1ev1aaatCvAUfKttLearuWrP9MDH5MBPbIqV92AaeXatLxBI9gBaebbnrfifHhDYfgasaacH8akY=wiFfYdH8Gipec8Eeeu0xXdbba9frFj0=OqFfea0dXdd9vqai=hGuQ8kuc9pgc9s8qqaq=dirpe0xb9q8qiLsFr0=vr0=vr0dc8meaabaqaciaacaGaaeqabaqabeGadaaakeaafaqabeqadaaabaGaeiyjauIaem4ta8Kaeyypa0ZaaSaaaeaacqWGUbGBaeaacqWGobGtaaGaey41aqRaeGymaeJaeGimaaJaeGimaadabaGaeiyjauIaemOta4Kaeyypa0ZaaSaaaeaacqWGobGtcqWGUbGBaeaacqWGobGtcqWGWbaCaaGaey41aqRaeGymaeJaeGimaaJaeGimaadabaGaeiyjauIaem4vaCLaeyypa0ZaaSaaaeaacqWGqbaucqWGWbaCaeaacqWGqbaucqWG0baDaaGaey41aqRaeGymaeJaeGimaaJaeGimaadaaaaa@516D@

where *n *= number of stomachs that have the prey *i*, *N *= total number of analyzed stomachs; *N*_*n *_= number of prey items of prey group n observed, *N*_*t *_= total number of prey items of all prey groups, *Pp *= weight of prey items of prey group *p *observed and *Pt *= total weight of prey items of all prey groups. The contribution of each prey to the diet was also estimated with the Index of Relative Importance (IRI) and its standardized value (%IRI) [28] as:

IRI=(%N+%W)×%O%IRI=100×IRIi∑IRIi
 MathType@MTEF@5@5@+=feaafiart1ev1aaatCvAUfKttLearuWrP9MDH5MBPbIqV92AaeXatLxBI9gBaebbnrfifHhDYfgasaacH8akY=wiFfYdH8Gipec8Eeeu0xXdbba9frFj0=OqFfea0dXdd9vqai=hGuQ8kuc9pgc9s8qqaq=dirpe0xb9q8qiLsFr0=vr0=vr0dc8meaabaqaciaacaGaaeqabaqabeGadaaakeaafaqabeqacaaabaGaemysaKKaemOuaiLaemysaKKaeyypa0ZaaeWaaeaacqGGLaqjcqWGobGtcqGHRaWkcqGGLaqjcqWGxbWvaiaawIcacaGLPaaacqGHxdaTcqGGLaqjcqWGpbWtaeaacqGGLaqjcqWGjbqscqWGsbGucqWGjbqscqGH9aqpdaWcaaqaaiabigdaXiabicdaWiabicdaWiabgEna0kabdMeajjabdkfasjabdMeajjabdMgaPbqaamaaqaeabaGaemysaKKaemOuaiLaemysaKKaemyAaKgaleqabeqdcqGHris5aaaaaaaaaa@515B@

where IRIi is the IRI value for each prey category of prey i.

The %O, %N and %W values of each prey were plotted following the method proposed by Cortés [28], which allows for an easy and adequate interpretation of prey importance in the diet of predators.

Differences in bathymetric distribution and diel (day-night) feeding activity were analyzed with a Kruskal-Wallis test [53]. For the analysis of feeding activity, the percentage by weight data of each stomach were grouped into four daily activity periods (morning, afternoon, night and dawn).

Diet breadth and overlap were estimated from %IRI values. Breadth of diet was calculated by using both Levins' index and its standardized form [54] as follows:

Bi=(1∑Pj2)(iv)andBA=(B−1n−1)(v)
 MathType@MTEF@5@5@+=feaafiart1ev1aaatCvAUfKttLearuWrP9MDH5MBPbIqV92AaeXatLxBI9gBaebbnrfifHhDYfgasaacH8akY=wiFfYdH8Gipec8Eeeu0xXdbba9frFj0=OqFfea0dXdd9vqai=hGuQ8kuc9pgc9s8qqaq=dirpe0xb9q8qiLsFr0=vr0=vr0dc8meaabaqaciaacaGaaeqabaqabeGadaaakeaafaqabeqafaaaaeaacqWGcbGqdaWgaaWcbaGaemyAaKgabeaakiabg2da9maabmaabaWaaSaaaeaacqaIXaqmaeaadaaeabqaaiabdcfaqnaaDaaaleaacqWGQbGAaeaacqaIYaGmaaaabeqab0GaeyyeIuoaaaaakiaawIcacaGLPaaaaeaacqGGOaakcqqGPbqAcqqG2bGDcqGGPaqkaeaacqqGHbqycqqGUbGBcqqGKbazaeaacqWGcbGqdaWgaaWcbaGaemyqaeeabeaakiabg2da9maabmaabaWaaSaaaeaacqWGcbGqcqGHsislcqaIXaqmaeaacqWGUbGBcqGHsislcqaIXaqmaaaacaGLOaGaayzkaaaabaGaeiikaGIaeeODayNaeiykaKcaaaaa@4F5B@

where *B*_*i *_= Levins' measure of niche breadth, *P*_*j *_= proportion of diet of predator that is made up of prey *j*, *B*_*A *_= Levins' standardized niche breadth and *n *= number of prey categories. Levins' index of niche breadth (*B*_*i*_) ranges from 1 to *n*, whereas, Levins' standardized niche breadth (*B*_*A*_) ranges from 0 to 1; low values indicate diets dominated by few prey items (specialist predators) while higher values indicate generalist diets [54].

A numerical abundance dietary matrix of prey items was constructed to calculate diet overlap between elasmobranch species by using the Pianka index [54] as follows:

Ojk=∑inPijPik∑inpij2∑inpik2
 MathType@MTEF@5@5@+=feaafiart1ev1aaatCvAUfKttLearuWrP9MDH5MBPbIqV92AaeXatLxBI9gBaebbnrfifHhDYfgasaacH8akY=wiFfYdH8Gipec8Eeeu0xXdbba9frFj0=OqFfea0dXdd9vqai=hGuQ8kuc9pgc9s8qqaq=dirpe0xb9q8qiLsFr0=vr0=vr0dc8meaabaqaciaacaGaaeqabaqabeGadaaakeaacqWGpbWtcqWGQbGAcqWGRbWAcqGH9aqpdaWcaaqaamaaqahabaGaemiuaa1aaSbaaSqaaiabdMgaPjabdQgaQbqabaGccqWGqbaudaWgaaWcbaGaemyAaKMaem4AaSgabeaaaeaacqWGPbqAaeaacqWGUbGBa0GaeyyeIuoaaOqaamaakaaabaWaaabCaeaacqWGWbaCdaqhaaWcbaGaemyAaKMaemOAaOgabaGaeGOmaidaaOWaaabCaeaacqWGWbaCdaqhaaWcbaGaemyAaKMaem4AaSgabaGaeGOmaidaaaqaaiabdMgaPbqaaiabd6gaUbqdcqGHris5aaWcbaGaemyAaKgabaGaemOBa4ganiabggHiLdaaleqaaaaaaaa@536C@

where O_*jk *_= Pianka measure of niche overlap between species j and species k ; and *P*_*ij *_and *P*_*ik *_= proportions of predator *j *and *k *with prey *i *in their stomachs. Diet overlap increases as the Pianka index increases and overlap is generally considered to be biologically significant when the value exceeds 0.60 [29].

To evaluate the statistical significance of estimated overlaps, observed overlap values were compared with a distribution of expected values based on simulations of a null model. The distribution of this model was realized with 1000 repetitions using the random algorithm R3. The observed values were considered statistically different from the null distribution values if they were higher or lower than 95% of the simulated indices [30].

## Authors' contributions

AFN conceived the study, conducted the *in situ *work and measurements, analyzed and interpreted the data and wrote the first draft of manuscript. PAM participated in the *in situ *work, analyzed the data and contributed to the critical review of the draft. AG analyzed the data and contributed to the critical review of the draft. All authors assisted in writing the manuscript and approved the final version.
